# Supplementary material for: Objective Assessments of Smoking and Drinking Outperform Clinical Phenotypes in Predicting Variance in Epigenetic Aging
Source: Genes (Basel). 2024 Jul 2;15(7):869. doi: 10.3390/genes15070869 (PMC11276345; doi:10.3390/genes15070869)
Supplement: Supplementary file 1 [file genes-15-00869-s001.zip › genes-3077967-supplementary.pdf]

Table Supplementary Table S1.
Clinical and Demographic Characteristic of Participants using pairwise deletion.

	Observations	Female	Male
Sex	314	186	128
Age	314	45.7 ± 7	49.3 ± 9.2
**Physiologic Parameters**			
BMI**	310	35.4 ± 8.6	32 ± 7.9
Systolic BP**	310	128 ± 21 mm Hg	140 ± 24 mm Hg
Diastolic BP**	310	87 ± 12 mm Hg	87 ± 14 mm Hg
Cholesterol	310	182 ± 39 mg/dL	179 ± 42 mg/dL
LDL**	303	106 ± 34 mg/dL	102 ± 39 mg/dL
HDL**	311	54 ± 22 mg/dL	48 ± 14 mg/dL
HbA1c**	306	5.9 ± 1.5%	6.4 ± 1.7%
Triglycerides**	311	118 ± 76 mg/dL	153 ± 112 mg/dL
**Self-Reported Behaviors and Conditions**			
Smoking	314	32 (17%)	33 (31%)
Binge Drinking	314	39 (21%)	44 (34%)
Heart Disease	314	20 (11%)	18 (14%)
Hypertension	314	100 (54%)	70 (55%)
Diabetes	314	33 (18%)	27 (21%)
Arthritis	310	56 (30%)	25 (20%)
Cancer	310	4 (2%)	3 (2%)
Liver Disease	310	4 (2%)	1 (1%)
Kidney Disease	310	8 (4%)	6 (5%)
Cataracts	310	7 (4%)	9 (7%)
**Exposure to Community Crime**			
Crime	310	.110 ± .26	.187 ± .31
**Epigenetic Measures of Age and Aging**			
GrimAge	314	52.6 ± 8.7 years	58.5 ± 8.4 years
GrimAge2	314	58.9 ± 7.3 years	64 ± 8.7 years
PCGrimAge	314	60 ± 6.2 years	65.9 ± 7.8 years
GrimAgeAcc*	314	6.9 ± 7.6 years	9.2 ± 5.7 years
GrimAge2Acc*	314	13.2 ± 5.9 years	14.7 ± 6.5 years
PCGrimAge Acc*	314	14.3 ± 4.1 years	16.6 ± 4.4 years
DunedinPace	314	1.07 ± 0.16	1.07 ± 0.14
Dcg05575921	303	79 ± 16%	69 ± 21%
ATS	306	1.5 ± 3.1	3.2 ± 3.8
Cg19693031	314	78.1%	73.1%

Note: BMI = Body Mass Index; Systolic BP = Systolic Blood Pressure; Diastolic BP = Diastolic Blood Pressure; LDL = Low-Density Lipoprotein; HDL = High-Density Lipoprotein; HbA1c = Glycated Hemoglobin; Triglycerides = Triglycerides are a type of fat found in the blood. GrimAgeAcc = Acceleration of Grim age is determined by subtracting chronological age from methylation-based age. GrimAge2Acc = Grim Age 2 Based on Real Age is determined by subtracting chronological age from methylation-based age. PCGrimAge Acc = Principal component Grim Age acceleration is determined by subtracting chronological age from methylation-based age.

Supplementary Table S2

The correlation of all study variables in this study (N=278).

	1	2	3	4	5	6	7	8	9	10	11	12	13	14	15
1. GrimAge	—														
2. GrimAge2	.95**	—													
3. PCGrimAge	.88**	.92**	—												
4. GrimAcc	.51**	.38**	.14*	—											
5. Grim2Acc	.38**	.34**	.10	.91**	—										
6. PCGrimAcc	.25**	.23**	.12	.81**	.86**	—									
7. PACE	.05	.30**	.26**	-.12	.19**	.15*	—								
8. Age	.67**	.73**	.86**	-.29**	-.36**	-.41**	.16**	—							
9. Sex	.33**	.32**	.40**	.17**	.15*	.30**	.04	.21**	—						
10.BMI	-.22**	-.15*	-.23**	-.12*	-.02	-.14*	.22**	-.14*	-.20**	—					
11.Systolic	.19**	.21**	.22**	.07	.08	.09	.10	.15*	.27**	.05	—				
12. Diastolic	.00	.02	-.02	.04	.08	.03	.08	-.03	.01	.17**	.71**	—			
13. CLT	-.02	-.05	.02	-.10	-.15*	-.09	-.11	.06	-.04	-.11	.10	.09	—		
14. LDL	-.07	-.07	-.02	-.13*	-.14*	-.10	-.01	.03	-.06	-.02	.12*	.13*	.91**	—	
15. HDL	-.03	-.09	-.07	-.02	-.10	-.09	-.29**	-.02	-.15*	-.18**	-.08	.00	.37**	.16**	—
16. HbA1c	.08	.16**	.15*	-.02	.07	.05	.21**	.10	.15*	.12	.23**	.09	-.05	-.03	-.22**
17. Trigly	.17**	.17**	.16**	.12*	.13*	.14*	.10	.08	.19**	-.03	.08	-.05	.17**	-.04	-.26**
18. Smoking	.26**	.27**	.19**	.44**	.48**	.51**	.13*	-.09	.15*	-.18**	.09	.03	-.02	-.02	-.05
19. Binge	.08	.05	.05	.196**	.17**	.24**	-.06	-.08	.16**	-.14*	.04	.02	.03	-.05	.14*
20. Heart	.24**	.31**	.26**	.06	.13*	.05	.22**	.22**	.09	.13*	.06	-.02	-.18**	-.14*	-.11
21. HPTS	.18**	.23**	.25**	-.17**	-.15*	-.22**	.12*	.34**	.02	.21**	.26**	.22**	-.03	.00	-.15*
22. Diabetes	.06	.12*	.12	-.05	.00	-.02	.16**	.12	.05	.11	.01	-.12*	-.12*	-.12	-.17**
23. Arthritis	.169**	.19**	.21**	-.12*	-.12*	-.17**	.09	.28**	-.11	.21**	.03	-.03	.08	.13*	-.04
24. Cancer	.22**	.23**	.26**	.01	-.01	-.04	.05	.24**	.05	-.04	.01	-.01	.03	-.01	.01
25. Liver	.24**	.25**	.24**	-.03	-.05	-.13*	.01	.29**	-.04	-.07	.04	.00	.07	.03	.10
26. Kidney	.17**	.20**	.19**	-.03	-.02	-.09	.10	.22**	.02	.01	.00	-.07	-.15*	-.15*	-.02
27. Cataracts	.23**	.24**	.30**	-.07	-.11	-.09	-.02	.32**	.06	-.14*	.07	-.05	-.13*	-.13*	.00
28. Crime	.12*	.11	.07	.18**	.18**	.17**	.08	-.02	.13*	.05	.00	.03	.02	.06	-.03
29. Dcg055	-.37**	-.38**	-.34**	-.53**	-.57**	-.68**	-.20**	.04	-.30**	.28**	-.09	.02	-.04	-.02	-.03
30. ATS	.53**	.60**	.57**	.40**	.48**	.54**	.33**	.25**	.24**	-.24**	.15*	-.03	-.04	-.10	-.03
31. cg1969	-.24**	-.19**	-.18**	-.24**	-.18**	-.20**	.08	-.06	-.31**	.08	-.14*	.01	-.10	-.01	.00

Supplementary Table S2 Continued
The correlation of all study variables in this study (N=278).

	16	17	18	19	20	21	22	23	24	25	26	27	28	29	30	31
16. HbA1c	—															
17. Trigly	.24**	—														
18. Smoking	-.08	.04	—													
19. Binge	-.08	.07	.29**	—												
20. Heart	.12*	-.03	.11	-.07	—											
21. HPTS	.19**	.08	-.02	-.03	.24**	—										
22. Diabetes	.56**	.16**	-.09	-.09	.14*	.22**	—									
23. Arthritis	.07	-.07	-.01	-.05	.11	.20**	.14*	—								
24. Cancer	.02	.09	-.02	.01	.04	.07	.21**	.04	—							
25. Liver	-.03	.00	.03	-.06	.15*	.11	.09	.06	.44**	—						
26. Kidney	-.06	-.03	-.10	-.05	.39**	.18**	.23**	.04	.11	.13*	—					
27. Cataracts	-.02	-.04	-.01	-.05	.16**	.12*	.12*	.14*	.09	.10	.36**	—				
28. Crime	-.04	-.03	.08	.10	-.03	.04	-.04	.05	-.05	-.04	.01	-.05	—			
29. Dcg055	.05	-.07	-.69**	-.27**	-.03	.16**	.11	.07	-.03	-.04	.12*	.02	-.11	—		
30. ATS	.12*	.18**	.43**	.13*	.18**	.02	.13*	.02	.11	.09	.12*	.18**	.05	-.59**	—	
31. cg1969	-.44**	-.29**	-.12	-.07	-.07	-.06	-.22**	.09	-.07	.09	.08	.02	.07	.10	-.17**	—

Note: PCGrim6acc = Principal component Grim Age acceleration; GrimAcc = Grim Age acceleration; Grim2Acc = Accelerated Grim Age 2; PACE = DunedinPACE; BMI = Body Mass Index; Systolic = Systolic Blood Pressure; Diastolic = Diastolic Blood Pressure; CLT = Cholesterol; LDL = Low-Density Lipoprotein; HDL = High-Density Lipoprotein; HbA1c = Glycated Hemoglobin; Trigly = Triglycerides; Binge = Binge Drinking; Heart = Heart Disease; HPTS = Hypertension; Liver = Liver Disease; Kidney = Kidney Disease; Crime = Neighborhood Crime; Dcg055 = Dcg05575921; cg1969 = cg19693031.
† $p < 0.1$, * $p < 0.05$, ** $p < 0.01$

Supplementary Table S3. **Regression Modeling predicting PCGrimAge Accelerated Aging using pairwise deletion**

		Observations	Adj. R2	AIC	BIC
**Demographic**	Age	314	**0.171**	1762	1770
	Sex	314	**0.064**	1800	1808
**Epigenetic**	Dcg05575921 (Dcg055)	303	**0.478**	1556	1564
	ATS	306	**0.290**	1664	1671
	cg19693031	314	**0.036**	1809	1817
**Vitals**	BMI	310	**0.026**	1791	1799
	Systolic	310	0.008	1797	1805
	Diastolic	310	-0.000	1800	1807
**Serum**	HbA1c	306	-0.003	1763	1770
	Cholesterol	310	0.002	1800	1808
	LDL	303	0.006	1759	1766
	HDL	311	0.005	1804	1812
	Triglycerides	311	**0.015**	1801	1808
**Med History**	Smoking	314	**0.279**	1781	1726
	Binge Drinking	314	**0.057**	1804	1811
	Heart Disease	314	-0.002	1821	1829
	Hypertension	314	**0.037**	1809	1817
	Diabetes	314	-0.003	1822	1829
	Arthritis	310	**0.021**	1789	1796
	Cancer	310	-0.003	1796	1804
	Liver Disease	310	**0.010**	1792	1800
	Kidney Disease	310	-0.002	1796	1803
	Cataracts	310	**0.009**	1792	1800
Crime	Crime	310	**0.038**	1783	1791
**Model**					
1	Age + Sex	314	**0.297**	1711	1723
2	Dcg055 + ATS	303	**0.509**	1539	1550
3	Age + Sex +Dcg055 + ATS	303	**0.751**	1335	1354
4	Model 3 + cg19693031	303	0.753	1334	1357
5	Model 3 + BMI	299	0.755	1316	1338
6	Model 3 + Systolic	299	0.754	1317	1340
7	Model 3 + HDL	300	**0.758**	1317	1339
8	Model 3 + Triglycerides	300	0.754	1322	1344
9	Model 3 + Smoking	303	0.750	1337	1360
10	Model 3 + Binge Drinking	303	0.750	1337	1360
11	Model 3 + Hypertension	303	0.750	1337	1359
12	Model 3 + Arthritis	299	0.748	1320	1342
13	Model 3 + Liver Disease	299	0.747	1321	1344
14	Model 3 + Cataracts	299	0.747	1322	1344

15	Model 3 + crime	299	**0.751**	1317	1339
16	Model 3 + all significant predictors*	296	**0.757**	1298	1324
17	Model 3 + all significant predictors *+Pace	296	0.757	1300	1330
18	Model 3 + Pace	303	0.752	1335	1357

Univariate regression results whose significance is p<0.05 values are indicated by bolding. AIC = Akaike's information criterion; BIC= Bayesian Information Criterion; * = HDL and Crime; PACE = DunedinPACE.

Supplementary Table S4. Regression Modeling predicting DunedinPACE using pairwise deletion

		Observations	Adj. R2	AIC	BIC
**Demographic**	Age	314	**0.016**	-294	-287
	Sex	314	-0.003	-287	-280
**Epigenetic**	Dcg05575921 (Dcg055)	303	**0.045**	-285	-278
	ATS	306	**0.104**	-423	-415
	cg19693031	314	0.005	-290	-282
Vitals	BMI	310	**0.035**	-294	-286
	Systolic	310	0.006	-285	-277
	Diastolic	310	0.005	-284	-277
**Serum**	HbA1c	306	**0.033**	-292	-284
	Cholesterol	310	**0.011**	-287	-280
	LDL	303	-0.003	-275	-267
	HDL	311	**0.067**	-307	-299
	Triglycerides	311	-0.002	-285	-277
**Med History**	Smoking	314	**0.018**	-294	-286
	Binge Drinking	314	-0.002	-288	-280
	Heart Disease	314	**0.049**	-304	-297
	Hypertension	314	**0.013**	-292	-285
	Diabetes	314	**0.019**	-294	-287
	Arthritis	310	0.004	-284	-276
	Cancer	310	0.003	-284	-276
	Liver Disease	310	-0.002	-282	-275
	Kidney Disease	310	**0.012**	-286	-279
	Cataracts	310	-0.003	-282	-274
**Psychosocial**	Crime	310	0.005	-284	-277
**Model**					
1	Age	314	**0.020**	-294	-287
2	Dcg055 + ATS	303	**0.114**	-307	-296
3	Age + Dcg055 + ATS	303	**0.119**	-307	-293
4	Model 3+ BMI	298	**0.221**	-337	-319
5	Model 3 + HbA1c	294	**0.134**	-303	-285
6	Model 3 + Cholesterol	298	**0.134**	-307	-288
7	Model 3 + HDL	299	**0.193**	-329	-311
8	Model 3 + Smoking	303	0.116	-305	-387
9	Model 3 + Heart Disease	303	**0.144**	-315	-396
10	Model 3 + Hypertension	303	0.127	-309	-290
11	Model 3 + Diabetes	303	**0.128**	-309	-291
12	Model 3 + Kidney Disease	298	0.119	-300	-282
13	Model 3 + Crime	298	0.115	-299	-281
14	Model 3 + all significant predictors*	284	**0.261**	-334	-298
15	Model 3 + all significant + PCGrimAgeAcc	284	0.252	-320	-265

16	Model 3 + PCGrimAgeAcc	303	0.118	-306	-288

Univariate regression results whose significance is p<0.05 values are indicated by bolding. AIC = Akaike's information criterion; BIC= Bayesian Information Criterion; * = BMI, HbA1c, Cholesterol , HDL, Heart Disease, Diabetes; PCGrimAgeAcc = Principal component Grim Age acceleration.
